# Intelligent prediction modeling for flexural capacity of FRP-strengthened reinforced concrete beams using machine learning algorithms

**DOI:** 10.1016/j.heliyon.2023.e23375

**Published:** 2023-12-07

**Authors:** Majid Khan, Adil Khan, Asad Ullah Khan, Muhammad Shakeel, Khalid Khan, Hisham Alabduljabbar, Taoufik Najeh, Yaser Gamil

**Affiliations:** a COMSATS University Islamabad, Abbottabad Campus, 22060, Pakistan; b Department of Civil and Structural Engineering, University of Bradford, Bradford, West Yorkshire, BD7 1DP, UK; c Department of Civil Engineering, University of Engineering and Technology, Peshawar, 25120, Pakistan; d Department of Civil Engineering, College of Engineering in Al-Kharj, Prince Sattam Bin Abdulaziz University, Al-Kharj 11942, Saudi Arabia; e Operation, Maintenance, and Acoustics, Department of Civil, Environmental and Natural Resources Engineering, Lulea University of Technology, Sweden; f Department of Civil Engineering, School of Engineering, Monash University Malaysia, Jalan Lagoon Selatan, 47500 Bandar Sunway, Selangor, Malaysia

**Keywords:** Fiber-reinforced polymers, Flexural capacity, Machine learning, Gene expression programming, Multi-expression programming

## Abstract

Fiber-reinforced polymers (FRP) are widely utilized to improve the efficiency and durability of concrete structures, either through external bonding or internal reinforcement. However, the response of FRP-strengthened reinforced concrete (RC) members, both in field applications and experimental settings, often deviates from the estimation based on existing code provisions. This discrepancy can be attributed to the limitations of code provisions in fully capturing the nature of FRP-strengthened RC members. Accordingly, machine learning methods, including gene expression programming (GEP) and multi-expression programming (MEP), were utilized in this study to predict the flexural capacity of the FRP-strengthened RC beam. To develop data-driven estimation models, an extensive collection of experimental data on FRP-strengthened RC beams was compiled from the experimental studies. For the assessment of the accuracy of developed models, various statistical indicators were utilized. The machine learning (ML) based models were compared with empirical and conventional linear regression models to substantiate their superiority, providing evidence of enhanced performance. The GEP model demonstrated outstanding predictive performance with a correlation coefficient (R) of 0.98 for both the training and validation phases, accompanied by minimal mean absolute errors (MAE) of 4.08 and 5.39, respectively. In contrast, the MEP model achieved a slightly lower accuracy, with an R of 0.96 in both the training and validation phases. Moreover, the ML-based models exhibited notably superior performances compared to the empirical models. Hence, the ML-based models presented in this study demonstrated promising prospects for practical implementation in engineering applications. Moreover, the SHapley Additive exPlanation (SHAP) method was used to interpret the feature's importance and influence on the flexural capacity. It was observed that beam width, section effective depth, and the tensile longitudinal bars reinforcement ratio significantly contribute to the prediction of the flexural capacity of the FRP-strengthened reinforced concrete beam.

## 1 Introduction

Recently, the deterioration of structural components like buildings and bridges due to aging, material degradation, inadequate maintenance, and earthquakes have spurred researchers to develop effective and cost-efficient methods for repairing these structures. Various approaches are available to rehabilitate deteriorated structures, including the replacement of degraded members, externally bonded steel plates, adding supplementary elements, external post-tensioning, and concrete jackets or using steel [1,2]. While these conventional repair techniques can enhance the stiffness and strength of undesirable concrete building structures, they often result in increased dead load and need substantial time for implementation. Consequently, there is a growing demand to search for alternative techniques or materials for repairing or reinforcing deficient structural concrete elements.

A viable alternative method involves the application of fiber-reinforced polymers (FRP) for the purpose of retrofitting RC structures. Recently, FRP strengthening has been rapidly adopted due to its notable benefits, especially in terms of efficiency and cost-effectiveness [3,4]. FRP composites have emerged as the preferred option for reinforcing structures owing to their lightweight characteristics, exceptional strength, resistance to corrosion, and long-lasting durability. With the ability to be conveniently fabricated into different shapes, FRP materials offer convenience in construction applications [[5], [6], [7]]. The advantages of FRP reinforcement extend beyond its mechanical properties, as it is increasingly considered a substitute or enhancement to traditional construction materials in various sectors. The accurate prediction and assessment of the strengthening effect in RC beams using FRP can be attributed to the utilization of two principal methods: externally bonded (EB) and near-surface-mounted (NSM) techniques [8,9]. In practical applications, the precise forecast of the capacity of RC beam strengthened by FRP FRP is of great significance for the comprehensive assessment of the strengthening effectiveness and design [10].

Estimating the flexural capacity (*M*_*u*_) of FRP-strengthened RC beams traditionally involves theoretical deductions and experimental studies [[11], [12], [13]]. However, these approaches are often time-consuming and prone to inherent errors due to assumptions and simplifications, such as the plane section assumption [14]. The plane section assumption simplifies stress analysis by focusing on in-plane stresses induced by loads while neglecting perpendicular stresses. In cases with significant out-of-plane stresses, a full 3D analysis may be needed for accuracy [15]. However, this simplification may introduce errors, primarily when substantial third-direction stresses result from in-plane loads [16]. Conversely, machine learning (ML) can enhance precision in handling such intricate and complex scenarios. Moreover, the lack of availability of actual data for validation restricts the generalizability of the models. As an alternative, finite-element simulation has been explored for prediction purposes [17,18]. However, accurately estimating numerous model parameters remains challenging, leading to prediction precision uncertainties. Hence, there persists an ongoing requirement to advance a precise and reliable technique for estimating flexural strength, aiming to enhance the accuracy and reliability of such predictions.

With the growth of soft computing methods, machine learning (ML) has emerged as a valuable tool in various applications in structural engineering. Its utilization spans multiple areas, including structural design and analysis, damage detection, the resistance of structural members under different actions, fire resistance of structures, structural health monitoring, and concrete's mechanical properties and mix design [[19], [20], [21]]. ML models have been employed to estimate collapse fragility, structural response demands, and performance metrics, among other factors, providing valuable insights into structural engineering for decision-making and optimization. The ability of ML algorithms to analyze large datasets and identify complex patterns has proven beneficial in improving the accuracy and efficiency of structural predictions and assessments. For instance, Mangalathu et al. [22] utilized ML methods to assess the shear strength of RC samples and their failure mechanism. Perera et al. [23] have established a neural network model for forecasting the shear strength of RC beams. Additionally, Mashrei et al. [24] introduced a back-propagation neural network (BNN) approach to estimate the strength of the bond between FRP and concrete joints. Naser [14] employed a combination of genetic algorithms and artificial neural networks (ANN) to assess the ultimate bending capacity of RC beams reinforced with FRP. Moreover, Abuodeh et al. [25] presented BNN model for the shear strength estimation of RC beams strengthened by reinforced FRP sheets externally. Amin et al. [26] utilized tree-based ensemble techniques to predict the flexural capacity of FRP-reinforced RC beams. In contrast to traditional empirical formulas and other approaches, ML-based techniques do not depend on assumptions based on physical or mathematical models. Instead, it learns patterns solely from experimental data. As a result, it effectively overcomes the limitations associated with conventional prediction methods. The findings consistently demonstrated that ML-based models consistently achieved higher levels of prediction accuracy compared to empirical models.

Numerous investigations have analyzed and proposed numerical models to anticipate the flexural response of FRP-reinforced bars concrete beams. However, many of these developed techniques tend to be intricate and complex. Recently, researchers have turned to ANN as an alternative approaches for predicting the behavior of RC components, particularly in situations where code standards are unavailable. Among these approaches, evolutionary algorithms such as GEP and MEP hold an advantage over ANN because they can construct precise prediction models even with a relatively small database [[27], [28], [29]]. However, despite these advancements, there is still a disagreement in the current literature concerning the development of a concise predictive mathematical equation that effectively and accurately estimates the flexural behavior of FRP-strengthened RC beams. Furthermore, the ANN technique is considered a black box, showing a lack of interpretability and explainability [30]. In contrast, the GEP and MEP algorithms are not black-box models because the internal mechanisms and operations are well-defined and understood [31,32]. They follow specific rules and genetic functions, such as mutation, selection, and crossover, which are explicitly programmed [28,33]. Researchers and practitioners can see how the algorithms evolve and generate solutions. In addition, SHAP-based interpretation was provided in this study to provide an enhanced explanation of the predictive developed models.

This research focuses on developing accurate and valid predictive models based on GEP and MEP techniques. For the model development, a total of 200 data points were utilized. The most significant input features were utilized for the development of both models. Various statistical parameters were utilized to assess the developed model's accuracy. The model's estimation performance was compared with traditional linear regression models and empirical estimation equations. Furthermore, the utilization of the Shapley Additive exPlanation (SHAP) technique was presented to provide interpretations regarding the significance and influence of input features on the flexural capacity RC beam.

## 2 Overview of ML algorithms

### 2.1 Gene expression programming

**Fig. 1:**
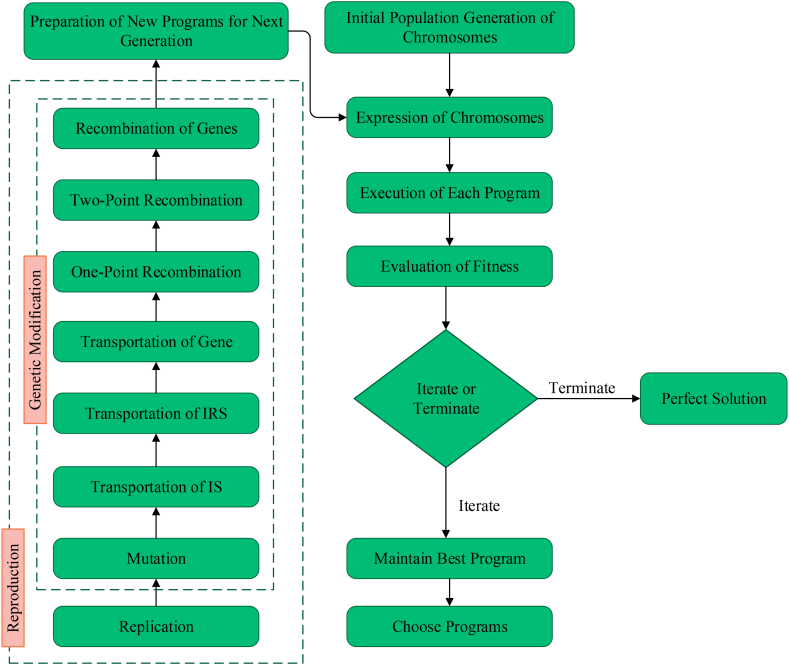
Flowchart of GEP technique.

Genetic algorithm (GA) is a type of evolutionary technique that utilizes a stochastic methodology to explore and enhance solutions for particular problems, drawing inspiration from genetic processes [34]. In 1985, Cramer presented genetic programming (GP), a technique that Koza expanded and refined to accommodate various sizes and configurations [35]. Later on, Ferreira introduced Gene Expression Programming (GEP) as a computer programming methodology based on the genotype-phenotype concept [36]. GEP employs a tree-like structure to construct intricate computer models capable of learning and evolving by modifying their shapes, sizes, and configurations, mirroring the adaptive characteristics observed in natural biological organisms. This approach, in accordance with Darwin's theory of evolution and the principles of Mendel's genetic theory, embodies the philosophy of computational intelligence [37]. GEP employs linear chromosomes with predetermined lengths to encode programs that address complex problems. One notable advantage of the GEP model is its potential to offer accurate predictions using straightforward mathematical equations, enhancing prediction efficiency [38]. Fig. 1 illustrates the comprehensive process flowchart of the GEP model.

**Fig. 2:**
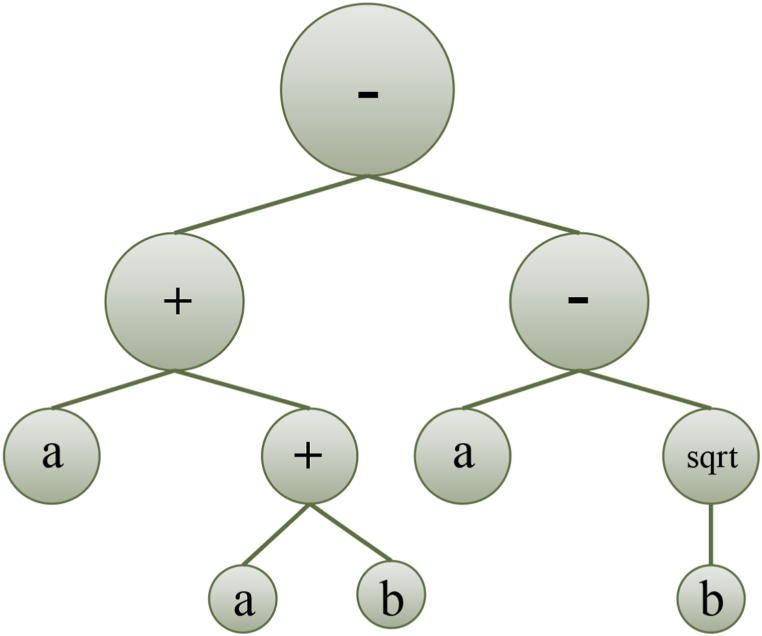
Chromosome expression tree.

A typical chromosome structure has a head section connecting terminal symbols or functions and a tail section connecting only terminal symbols. The operational mechanism of the GEP technique is depicted in Fig. 2. Referring to the process diagram, the estimation process in GEP starts with the generation of random chromosomes by representing defined values according to the Karva language. To solve a problem using GEP, four essential elements must be known: the terminal set (which encompasses input parameters and constants) and the function, GEP governing features (such as mutation, size of the population, and crossover), termination conditions, and the fitness function [39,40]. Garg et al. [41] suggested that the functioning mechanism of the GEP technique can be split into several phases.

**Fig. 3:**
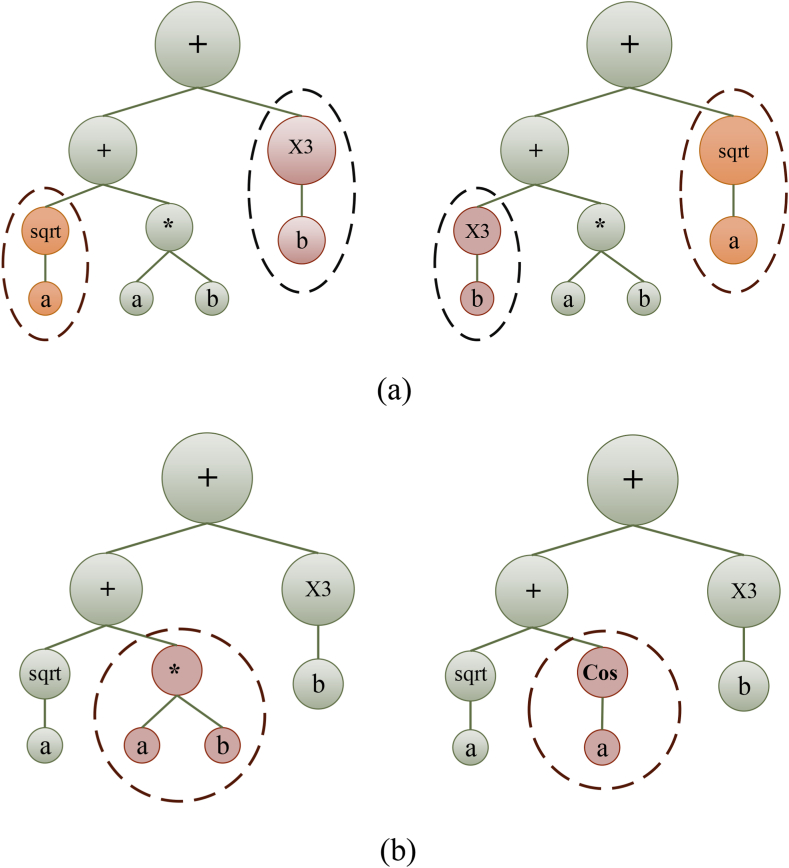
GEP chromosome tree with LISP language for (a) crossover and (b) mutation.

During the initialization phase, the population is generated according to the predefined function and terminal settings. The chromosomes can be readily transformed into algebraic equations, having fixed lengths [42]. Following that, the chromosomes undergo a transformation process into expression trees (ETs) of diverse shapes and sizes. The comprehension of the gene language syntax or pattern is analogous to understanding the language employed by expression trees (ETs) [36]. The genes can be combined through linking functions constituting the chromosome: subtraction, addition, division, and multiplication [43]. During the evolution phase, the generation of the population is assessed based on the criteria: the fitness function and the maximum number of iterations or generations. The decision to increase the number of expression trees (ETs) is determined by either indicating the occurrence of overfitting or meeting the termination condition of the iterative process. Various selection methods, including ranking selection, the roulette wheel approach, the elite strategic approach, greedy over-selection, and tournament selection, are utilized to identify viable chromosomes from the iteration and move them to the subsequent generation [44,45]. The program terminates if the population performance satisfies the desired criteria or if the highest number of iterations is attained. Conversely, if the termination condition is not satisfied, a new generation of the population is generated iteratively by employing three genetic functions: reproduction of chromosomes, crossover (illustrated in Fig. 3a), and mutation (depicted in Fig. 3b). This process continues until the threshold condition is met, leading to the acquisition of the optimal solution [46,47].

### 2.2 Multi expression programming

**Fig. 4:**
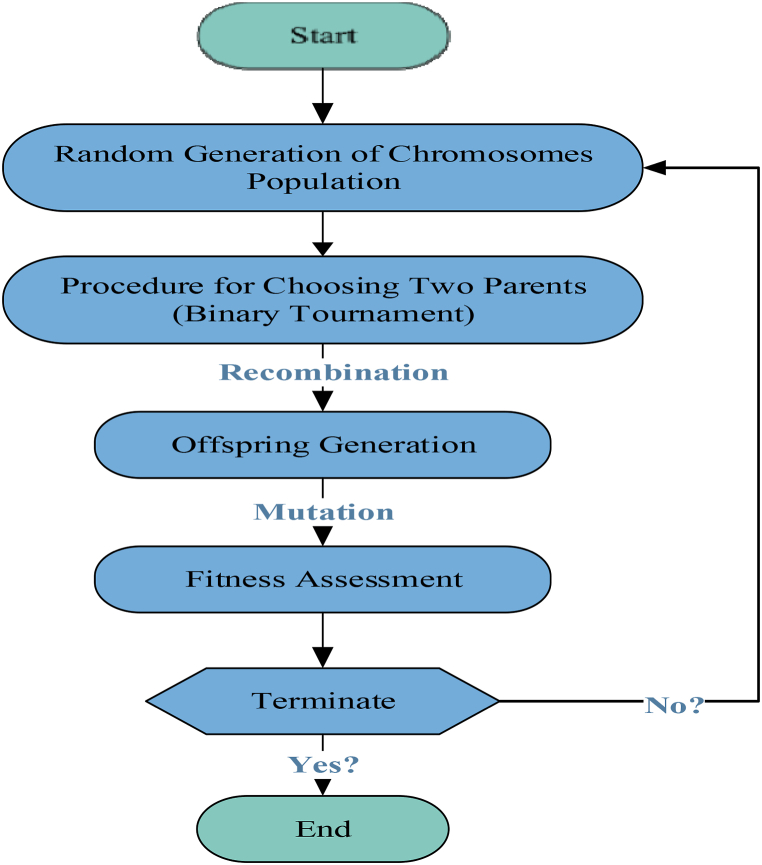
Process flowchart of MEP.

**Fig. 5:**
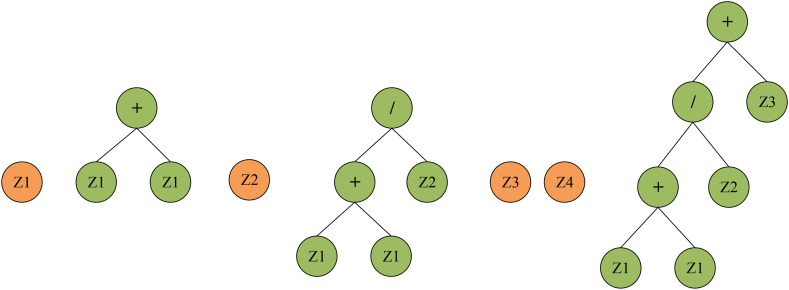
MEP architecture.

The MEP technique is a sophisticated form of linear GP that utilizes linear chromosomes to code solutions. The working operation of MEP, akin to that of GEP, is visually presented in Fig. 4, providing a clear illustration of its functioning. A distinctive characteristic of MEP, which sets it apart as a comparably recent branch of GP, is capable of coding multiple solutions within an exclusive chromosome. The optimum solution is subsequently determined by comparing the fitness values among these encoded programs [48,49]. Oltean and Grosan [50] employed the binary environment selection process; two parents are chosen and undergo recombination to generate two separate offspring. The iterative operation resumes until the termination criteria are satisfied, aiming to obtain the best program. Throughout this process, the offspring undergo mutation. Similar to GEP, the MEP model permits the adjustment of several parameters, such as the sub-population size and range, function set, crossover probability, and algorithm length. These parameters are crucial components in developing the MEP model [51]. Increasing sub-populations and their respective sizes in the MEP model results in more intricate evaluations and computationally demanding calculations, particularly population size, representing the total count of programs. Furthermore, the algorithm length notably affects the generated mathematical expression length. The architecture of the MEP model is visually illustrated in Fig. 5.

## 3 Methodology

### 3.1 FRP strengthening RC beam

**Fig. 6:**
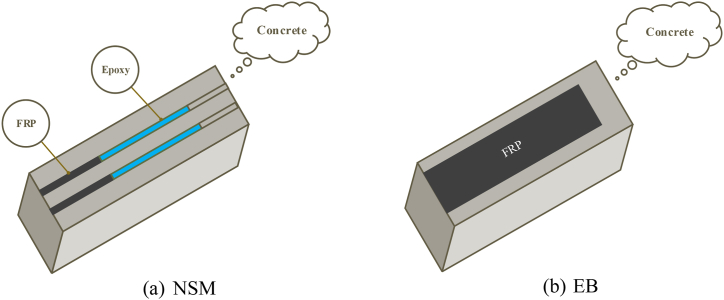
Strengthened RC beam with FRP (half span).

The primary methods for RC beam strengthening employing FRP are the NSM and EB methods, as shown in Fig. 6a and b, respectively. The EB method, which involves bonding FRP to the bottom of the beam, is the most commonly employed. It is relatively simple to implement but has a higher risk of FRP detachment, thereby reducing the FRP strength. In contrast, the NSM method provides enhanced utilization of FRP strength as the FRP is embedded within a groove at the beam bottom, reducing the likelihood of detachment. However, this method entails intricate procedures and may result in damage to the cover concrete beam. Both methods effectively augment the RC beam flexural strength by enabling the tensile reinforcement and FRP to bear the tensile forces jointly. Fig. 6 presents the layout of these two strengthening techniques, offering a visual representation of their arrangements.

### 3.2 Data collection

A robust and reliable prediction model relies on establishing a comprehensive database. This study incorporates the collection of experimental data pertaining to the reinforcement of rectangular RC beams utilizing FRP, which has been sourced from a variety of scholarly research findings [[7], [10], [11], [13], [52], [53], [54], [55], [56], [57], [58], [59], [60], [61], [62], [63], [64], [65], [66], [67], [68], [69], [70], [71], [72], [73], [74], [75], [76], [77], [78], [79], [80], [81], [82], [83], [84], [85], [86], [87], [88], [89], [90], [91], [92], [93], [94], [95], [96], [97], [98], [99], [100], [101], [102], [103], [104], [105], [106], [107], [108], [109], [110], [111], [112], [113], [114]] collected by Zhang et al. [115]. The database consists of 103 sets of EB data and 97 NSM data sets. To identify the key input features, the study considered the major characteristics influencing the flexural strength of FRP-strengthened RC beams.

**Fig. 7:**
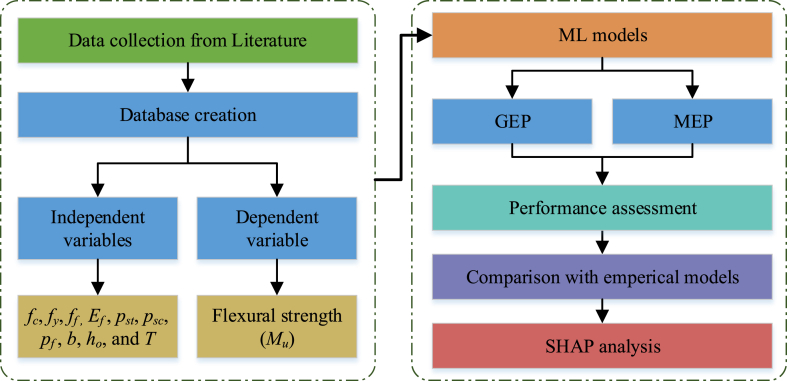
Methodology flow chart.

The selected input variables for the model included the beam width (*b*), FRP tensile strength (*f*_*f*_), section effective depth (*h*_*0*_), longitudinal bars tensile reinforcement ratio (*ρ*_*st*_), FRP plate/sheet/NSM ratio (*ρ*_*f*_), longitudinal bars compressive reinforcement ratio (*ρ*_*sc*_), FRP modulus (*E*_*f*_), concrete compressive strength (*f*_*c*_), reinforcement yield strength (*f*_*y*_), and strengthening method (*T*). Fig. 6 illustrates RC beams strengthened with FRP, covering half the span. The flexural capacity of the RC beam (*M*_*u*_*)* is considered as response parameter. Table 1 provides statistical evaluation for the input variables and output property (*M*_*u*_) in the FRP-strengthened RC beam data set. It presents each variable's standard deviation (SD), median, maximum, minimum, and mean values. The overall methodology followed in the current study is illustrated in Fig. 7.

**Table 1:** Statistical values of the data sets.

Statistics	Input variables										Output
	***f***_***c***_**(MPa)**	***f***_***y***_**(MPa)**	***f***f (**MPa)**	***E***_***f***_**(GPa)**	ρ_***st***_**(%)**	ρ_***sc***_**(%)**	ρ_***f***_**(%)**	***b* (mm)**	***h***_***o***_**(mm)**	***T***	***M***_***u***_**(kN.m)**
Mean	40.03	467.36	2379.41	150.05	0.81	0.43	0.23	161.73	225.14	1.48	48.44
Median	38.27	452	2400	159	0.69	0.33	0.16	150	222	1	41.01
Standard Error	0.84	7.01	85.24	4.94	0.03	0.02	0.02	3.46	3.86	0.04	2.26
Sample Variance	140.07	9830.92	1453254	4873.98	0.20	0.11	0.05	2397.42	2982.31	0.25	1025.56
Mode	40	414	2740	165	0.39	0.31	0.04	150	243	1	59.2
Standard Deviation	11.84	99.15	1205.51	69.81	0.45	0.33	0.23	48.96	54.61	0.50	32.02
Skewness	0.66	0.73	0.33	−0.34	1.83	2.30	2.67	2.41	0.09	0.06	1.18
Kurtosis	0.26	0.72	−0.38	−0.88	4.23	8.62	8.68	7.79	−0.36	−2.02	1.26
Minimum	17.5	242.2	512	22	0.23	0	0.02	100	118	1	5.79
Maximum	79.64	788	4950	271	2.58	2.58	1.46	500	370	2	160.2
Range	62.14	545.8	4438	249	2.35	2.58	1.44	400	252	1	154.41

**Fig. 8:**
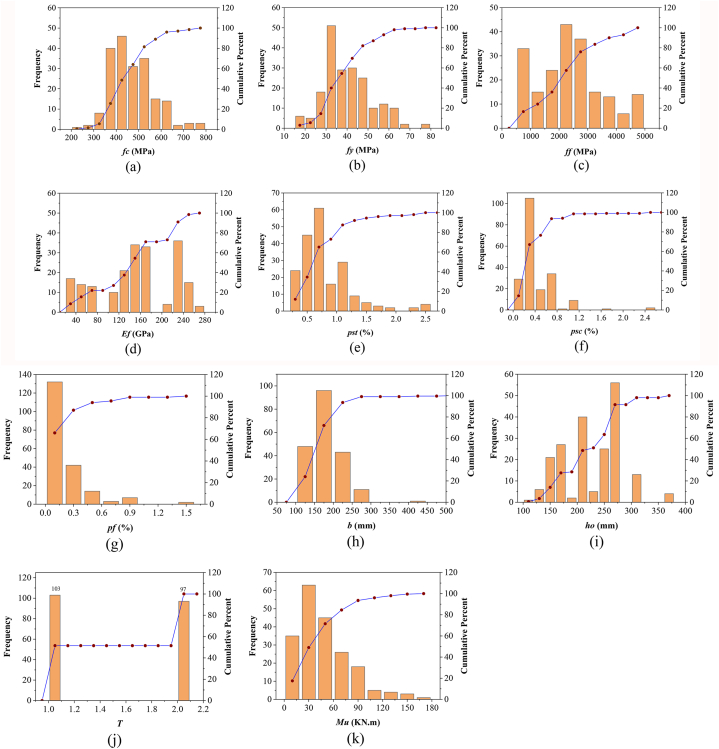
Frequency distribution histograms of inputs and input.

**Fig. 9:**
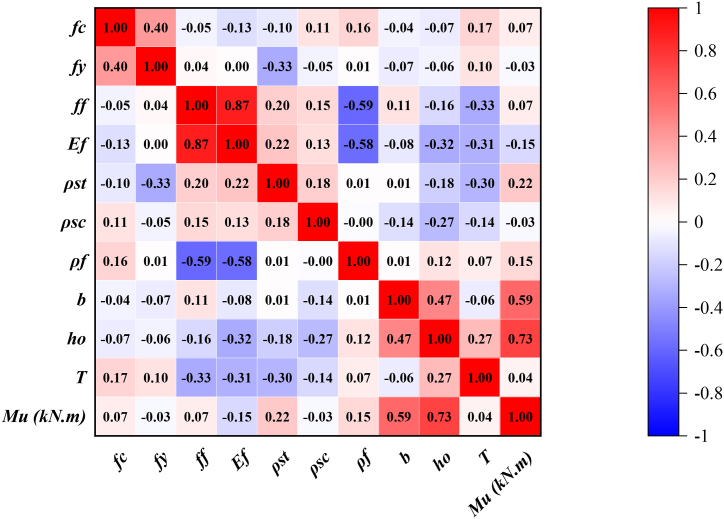
Correlation heat map of the variables.

The data values across the different variables in the obtained data exhibit a significantly wide range and dispersion. The data sets obtained were randomly partitioned into validation and training sets to facilitate the development of a robust model. The training set was employed to train the developed model, whereas the validation set was utilized to assess its effectiveness following the training phase. Consequently, the training set comprised 140 data points (70 %), and the validation set included 60 data points (30 %). To account for the diverse magnitudes and units of the input variables, the data within the training dataset underwent a normalization process prior to model training. This normalization process ensures smoother and easier convergence when seeking the optimal solution, thereby improving the performance and speed of the model. The frequency distribution histograms of variables are provided in Fig. 8a–k. In addition, correlation heat map is provided in Fig. 9. It can be noticed that flexural capacity has highest positive correlation (r) with effective depth (r = + 0.73) and beam width (r = +0.59).

### 3.3 Model development

Appropriate variable selection is a key step in the development of a robust prediction model. The GEP technique fitting parameters were decided by considering suggestions from prior research and conducting several test runs. The GEP model development encompasses three sets of fitting parameters: genetic operators, numerical constants, and general model parameters. The standard parameters encompass several aspects, including population size (representing the count of chromosomes), number of genes, set of functions, head size, and the connecting operator. The population size directly influences the duration of the process. This study utilized a population size of 1000 for GEP modeling. The head size serves as a regulator for the model's structure generated by the program. It determines the number of sub-ETs, while the gene count determines the complication of each term. In this study, the head size for the GEP model was set at 10, and the gene number was set at 4. Detailed descriptions of the GEP setting parameters, along with genetic operators, are provided in Table 2. The GEP-based algorithm was executed using GeneXpro Tool 5.0.

**Table 2:** Parameters setting of the GEP model.

Parameter	Setting
General	
Genes	4
Head size	10
Chromosomes	1000
Linking function	Multiplication
Set of functions	+, -, *,/, Exp, Ln, Inv, x^2^, x^3^, x^4^, x^5^, 3Rt, 6Rt
**Numerical constants**	
Constant per gene	10
Upper bound	10
Data type	Floating-point
Lower bound	−10
**Genetic operators**	
Mutation	0.00138
Permutation	0.00546
Inversion rate	0.00546
Random cloning	0.00102
IS transportation rat	0.00546
RIS transportation rate	0.00546
Gene transportation rate	0.00277
Recombination rate	0.00277
RNC mutation	0.00206
Dc mutation	0.00206

In MEP modeling, it is crucial to provide several MEP setup parameters to develop a robust model. These setup variables were set based on recommendations and multiple initial runs [116]. The population size determines the number of generated programs. A large population size results in a more complex and accurate model, but convergence takes longer. However, expanding the population size beyond a certain range can lead to overfitting the model [27]. Table 3 displays the selected setting variables for the developed MEP model. The available functions in the model include basic operators such as addition, division, multiplication, and subtraction. The generation number establishes the desired level of precision that the model should attain before the termination of the process. Running the algorithm for multiple generations helps to obtain a simulation model with the lowest errors. Different parameter combinations were tested to select the optimized model, ultimately choosing the combination that yielded the model with the smallest error values. Overfitting is a significant issue in machine learning modeling, referring to a situation where the model demonstrates strong performance on the original data but encounters difficulties when applied to new, unseen data. To mitigate this concern, it is suggested to assess the efficiency of the generated model on the unseen data, allowing for an evaluation of its generalization capabilities [117,118]. Accordingly, the data is divided into two categories. A separate validation set, which was not utilized in the developmental process of the model, was used to assess the effectiveness of the algorithm. The data was split into 70 % for training and 30 % for validation. The generated models demonstrated exceptional performance across all datasets. The MEPX v 1.0 software tool was employed to execute the MEP model development process.

**Table 3:** Setup for MEP model development.

Parameter	Setting
No. of subpopulations	50
Subpopulation size	250
Code length	50
Tournament size	2
Functions probability	0.5
Crossover probability	0.9
Mutation probability	0.01
Variables probability	0.5
Functions	+, -, *,/, Power, Sqrt, Exp, Pow10, Sin, Cos, Inv(1/x), ACos, Atan, Tan, ASin,

### 3.4 Model evaluation indicators

Several statistical parameters are employed to assess the validity and efficiency of the ML models, including R, MAE, RMSE, MSE, RRMSE, and the performance index (ρ), as expressed in Equations (1), (2), (3), (4), (5), (6), (7). It is essential to highlight that MSE, MAE, and RMSE are widely used statistical measures in machine learning to assess error levels [119]. Lower RMSE, MSE, and MAE values signify enhanced efficiency of the generated model.

(1) $$\text{RMSE}=\sqrt{\frac{\sum_{i=1}^{n}{\left(\text{ei}‐\text{mi}\right)}^{2}}{n}}$$

(2) $$\text{MAE}=\frac{\sum_{i=1}^{n}\left|\text{ei}‐\text{mi}\right|}{n}$$

(3) $$\text{RSE}=\frac{\sum_{i=1}^{n}{\left(\text{mi}‐\text{ei}\right)}^{2}}{\sum_{i=1}^{n}{\left(ē‐\text{ei}\right)}^{2}}$$

(4) $$R=\frac{\sum_{i=1}^{n}(\text{ei}‐ēi)\left(\text{mi}‐\;\bar{m}i\right)\;}{\sqrt{\sum_{i=1}^{n}{\left(\text{ei}‐\;ēi\right)}^{2}{\sum_{i=1}^{n}\left(\text{mi}‐\bar{m}i\right)}^{2}\;}}$$

(5) $$\text{RRMSE}=\frac{1}{\left|ē\right|}\;\sqrt{\frac{\sum_{i=1}^{n}{\left(\text{ei}‐\text{mi}\right)}^{2}}{n}}$$

(6) $$\rho =\frac{\text{RRMSE}}{1+R}$$

(7) $$\text{OF}=\left(\frac{n_{T}‐n_{v}}{n}\right)p_{T}+2\left(\frac{n_{v}}{n}\right)p_{v}$$

Where ei represents the ith original value, mi represents the ith forecasted value, and n indicates the number of data points. Furthermore, $\bar{m}i$ and ēi correspond to the mean of the predicted and actual experimental outcomes, respectively.

A greater value of the correlation coefficient (R) reveals the accuracy of the model. R quantifies the correlation between actual and model data [120]. R-value above 0.8 signifies a robust relation between the experimental and estimated values, indicating a strong relation between the two [121,122]. However, the R-value does not account for the impact of division, multiplication, or scaling by a fixed number on the output. Hence, R alone is insufficient to assess the overall efficiency of the model. RMSE and MAE compute the average magnitude of errors, each with significance. In RMSE, errors are squared prior to averaging, giving more weight to higher errors. A higher RMSE value reveals the need to minimize estimates with substantial errors. Conversely, MAE assigns relatively less importance to weighty errors and is consistently lower than RMSE. Furthermore, the generated model underwent external validation based on recommended standards, as detailed in Table 4.

**Table 4:** External validation conditions.

S. No	Expression	Conditions	Suggested by
**1**	$k=\frac{\sum_{i=1}^{n}\left(\text{ei}\times \text{mi}\right)}{{\text{ei}}^{2}}$	0.85<k<1.15	[123]
**2**	$k^{\prime }=\frac{\sum_{i=1}^{n}\left(\text{ei}\times \text{mi}\right)}{{\text{mi}}^{2}}$	0.85<k<1.15	
**3**	$R^{2}=1-\;\frac{\sum_{i=1}^{n}{\left(\text{mi}‐\;e_{i}^{{}^{\circ}}\right)}^{2}}{{\sum_{i=1}^{n}(\text{mi}‐\;m_{i}^{o})}^{2}}$ Where, $e_{i}^{{}^{\circ}}=k\times m_{i}$	$R^{2}≅1$	[124]
	$R_{{}^{\circ}}^{2}=1-\;\frac{\sum_{i=1}^{n}{\left(\text{ei}‐\;m_{i}^{{}^{\circ}}\right)}^{2}}{{\sum_{i=1}^{n}(\text{ei}‐\;e_{i}^{o})}^{2}}$ Where, $m_{i}^{{}^{\circ}}=k^{\prime }\times e_{i}$	$R_{{}^{\circ}}^{2}≅1$	
	$R_{m}=R^{2}\times \left(1‐\sqrt{|R^{2\;}‐R_{o}^{2}}|\right)$	$R_{m}>0.5$	
**4**	$m=\frac{R^{2}-{\;R}_{{}^{\circ}}^{2}}{R^{2}}$	m < 0.1	[125]

### 3.5 Interpretability of the models

SHAP is a methodology that originates from game theory and is employed to elucidate the effectiveness of an ML model. It creates an understandable model by adopting an additive attribute criterion approach, wherein the developed ML algorithm is represented as a linear combination of input features. In the context of a model with input parameters x = (x_1_, x_2_, …, x_p_), where p represents the input features number, and the explanation model g($x^{\prime }$) for the actual model f(x) is expressed by simplifying the input as $x^{\prime }$:

(8) $$f\left(x\right)=g\left(x^{\prime }\right)=\emptyset_{0\;}+\sum_{i=1}^{p}\emptyset_{i}x_{i}^{\prime }$$

**Fig. 10:**
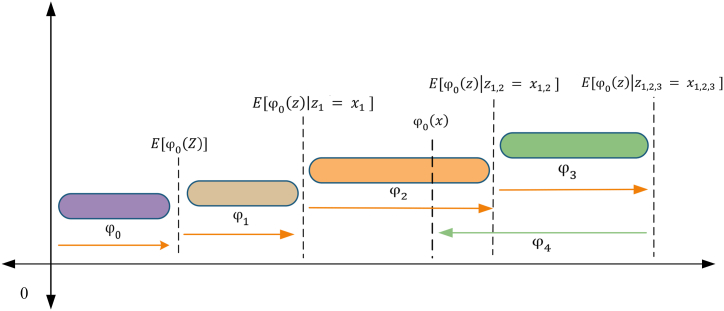
SHAP attributes.

In Equation (8), ∅_0_ denotes the constant value for all missing inputs, and p represents the number of input parameters. The correlation between the inputs $x^{\prime }$ and x is defined by the mapping function x = h_x_($x^{\prime }$). Fig. 10 illustrates Equation (8), where ∅_0_, ∅_1_, ∅_2_, and ∅_3_ increase the estimated value of g, while ∅_4_ lowers it. According to Lundberg and Lee [126], Equation (8) has a unique solution that exhibits desirable characteristics: consistency, local accuracy, and missingness. The principle of local accuracy necessitates the model to estimate an output corresponding to the combined feature attributions. This, in turn, demands that the model aligns with the output of function "f" when provided with the simplified input $x^{\prime }$. Missingness guarantees no importance is employed to missing attributes, which is satisfied when xi ′ = 0 implies ∅ = 0_i_. Consistency ensures that changing an attribute with a more significant effect will not lower the criterion allocated to that attribute. The graphical illustration of SHAP is shown in Fig. 10.

SHAP provides effective interpretations for machine learning models. Various methods can approximate SHAP values, including Tree SHAP, Kernel SHAP, and Deep SHAP. SHAP interaction values ensure consistent interpretations of relationship impacts for individual estimations. One notable advantage of SHAP values is their local and global interpretability. Unlike existing feature importance measures in ML techniques, SHAP can determine whether each input attribute contribution is negative or positive. Each data point can also have its corresponding SHAP value, allowing for the model's global and local interpretability. Various researchers in the literature also describe more comprehensive explanations of SHAP [126,127].

## 4 Results and discussion

**Fig. 11:**
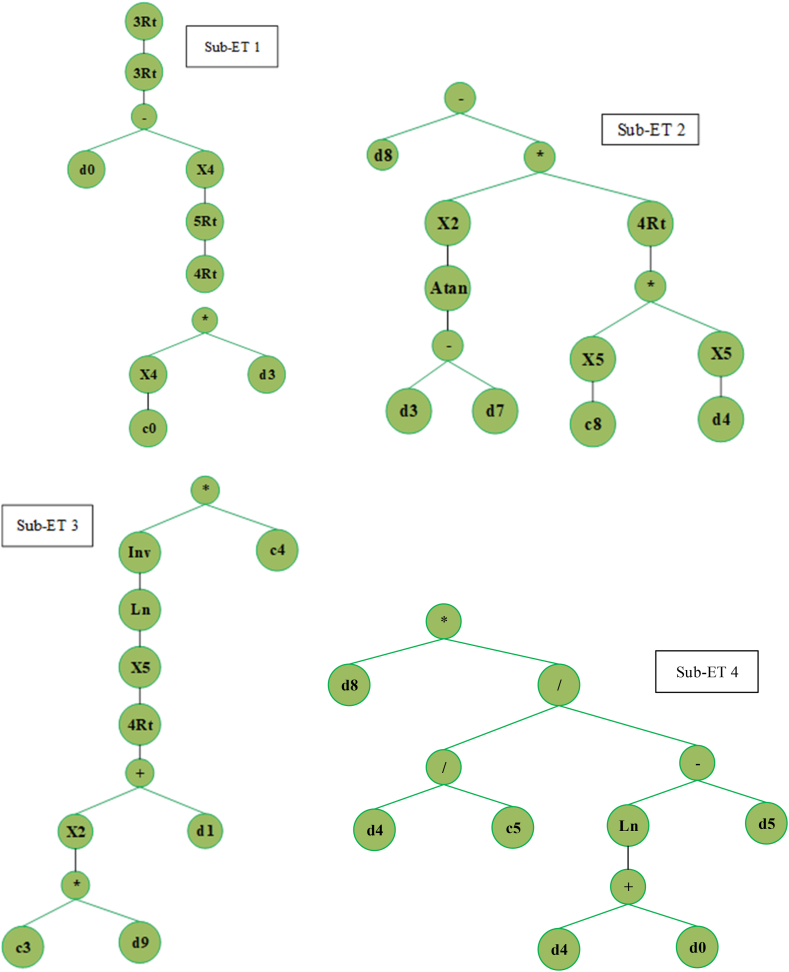
Expression trees of the GEP model.

The GeneXpro tool was employed to generate expression trees (ETs) for the proposed GEP algorithm, as illustrated in Fig. 11. Subsequently, these ETs were interpreted to derive an empirical formula for estimating the flexural capacity of the FRP-strengthened RC beam. This formulation utilizes fundamental arithmetic operators such as addition, subtraction, and multiplication, as well as mathematical functions such as +, -, *,/, Exp, ln, Inv, x2, x3, x4, x5, 3Rt, log, $\tan^{‐1}\text{,}$ and 6Rt. In addition, multiplication was employed as a linking function.

### 4.1 GEP formulation

Based on the expression trees, a simplified mathematical expression is formulated to estimate the flexural capacity of the FRP-strengthened RC beam, denoted by Equation (9). The proposed empirical formulation based on GEP has the potential to forecast the flexural capacity accurately.

(9) $$M_{u\;}=A\times B\times C\times D$$

Where,

$$A=\sqrt[{6}]{{\;f}_{c}\;‐\;1253.57E_{f}^{\;1/20}}$$

$$B=h_{o}\;‐\;16.45\times \rho_{st}^{5/4}\times \tan^{‐1}{\left(E_{f}\;‐\;b\right)}^{2}$$

$$C=\frac{1}{0.207\;\log\left(104.85T^{2}+{\;f}_{y}\right)}$$

$$D=\frac{h_{o}\rho_{st}}{6.553\left(\log\left(\rho_{st}+f_{c}\right)\;‐\;\rho_{sc}\right)}$$

### 4.2 Performance of models

This section provides an assessment of the model's efficiency, including statistical analysis, a comparison of regression slopes, and an evaluation of errors associated with the ML-generated models.

#### 4.2.1 Regression slope comparison

**Fig. 12:**
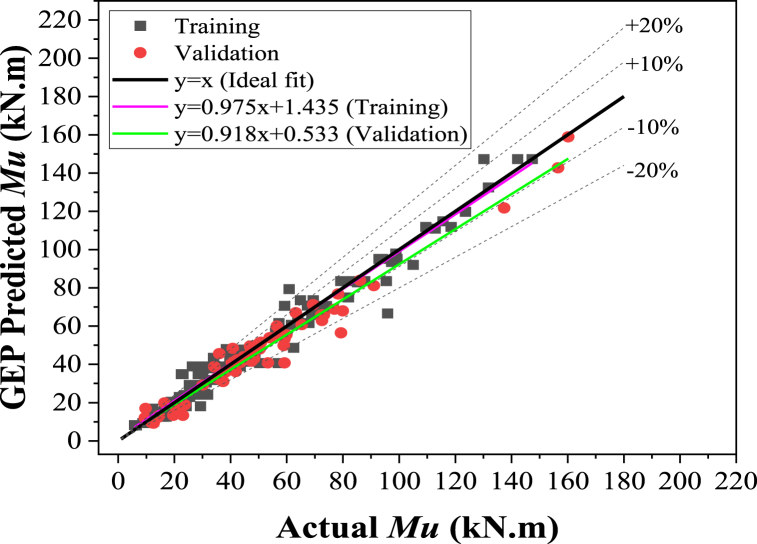
Comparison of actual ultimate flexure strength with GEP.

**Fig. 13:**
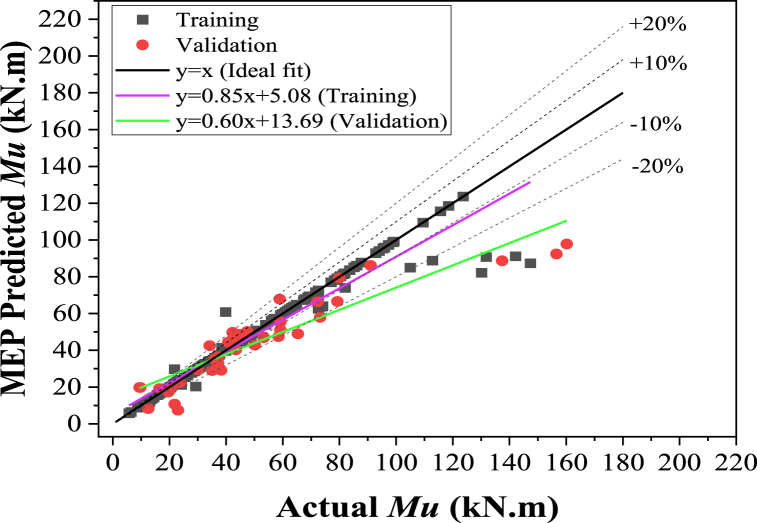
Comparison of Actual ultimate flexure strength with MEP.

This section evaluates the developed model's accuracy by analyzing the regression line slope generated from actual values plotted on the x-axis against estimated values on the y-axis, as shown in Fig. 12, Fig. 13. This approach is commonly used by researchers [28,38,128,129] to assess the precision of ML models. Fig. 12 shows that the developed GEP algorithm recorded regression slopes of 0.97 and 0.91 for the training and validation set, respectively. Similarly, from Fig. 13, these values were noted for the MEP model as 0.85 and 0.60 for the training and validation data sets, respectively. The GEP model demonstrated regression slopes greater than 0.8 for validation and training, indicating a superior correlation between actual and estimated values. Conversely, the MEP model exhibited a relatively lower slope for the validation set. Therefore, the GEP-developed model demonstrated more robustness than the MEP model.

#### 4.2.2 Error analysis

**Fig. 14:**
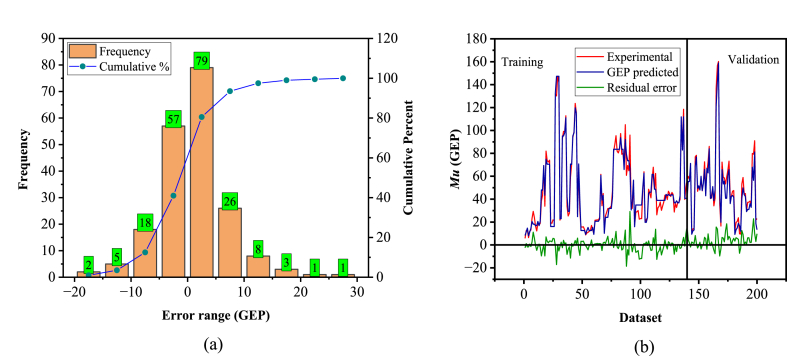
Experimental vs predicted values and error analysis for the GEP model.

**Fig. 15:**
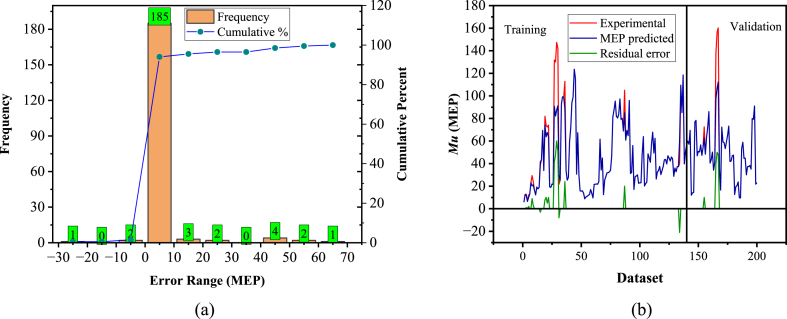
Experimental vs predicted values and error analysis for MEP model.

Fig. 14, Fig. 15 depict an error analysis of the generated model, presenting an error histogram and the actual results followed by the corresponding model estimations. This evaluation aligns with previous literature [38,130]. The error histograms in Fig. 14, Fig. 15a reveal that 90 % of the records lie between −10 and 10 (kN m) for the GEP model, while 92.5 % of the records fall within the range of 0–10 (kN m) for the MEP model. Fig. 14, Fig. 15b demonstrate that both models closely match the experimental results.

**Fig. 16:**
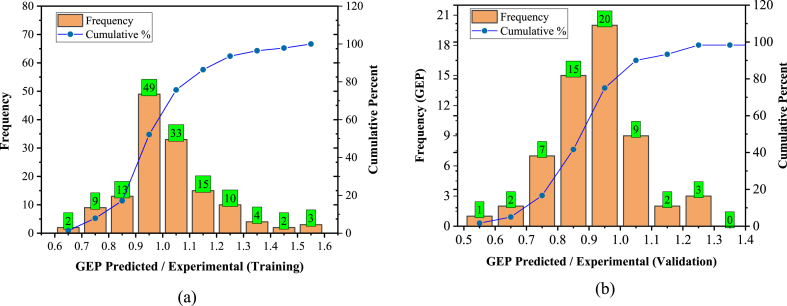
Predicted/Experimental ratio for GEP; (a) Training, (b) Validation.

**Fig. 17:**
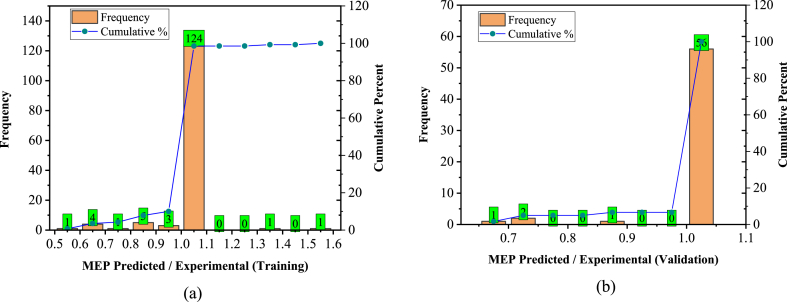
Predicted/Experimental ratio for MEP; (a) Training, (b) Validation.

Fig. 16, Fig. 17 present the model estimated-to-actual ratio for training and validation data sets, widely used as a statistical evaluation metric for AI models [131,132]. Notably, 110/140 (78.5 %) and 46/60 (76.6 %) prediction records fall within the ±20 % range of relative error for the training and validation data of the GEP model, respectively. Similarly, the MEP model exhibited 127/140 (90.7 %) and 56/60 (93.3 %) predicted values within the ±10 % range of relative error for training and validation, respectively.

#### 4.2.3 Statistical evaluation

Table 5 presents the performance indicators (MAE, RMSE, RRMSE, RSE, R, ρ, OF) of the proposed models. The value of R for both the generated models exceeds 0.96 for both validation and training datasets, with the GEP outperforming the MEP algorithm. Furthermore, most of the statistical error values of the GEP model are lower than those of MEP for both datasets. These lower values indicate the superior performance of both models, particularly the GEP approach. Additionally, the ρ values of the generated GEP model are 0.062 for training and 0.074 for validation, compared to the values obtained with MEP, which are 0.097 and 0.108 for training and validation, respectively. While OF values for the GEP and MEP models are 0.0695 and 0.1041. Consistently, the GEP model demonstrated superior efficiency in terms of RRMSE, RSE, and RMSE compared to the MEP model for both sets. These results validate the robustness of the GEP approach. Furthermore, the models are validated using external validation criteria. The proposed GEP and MEP model satisfied the external validation conditions, as shown in Table 6.

**Table 5:** Performance evaluation of the ML-based developed models.

Model	Subset	MAE	RMSE	RSE	RRMSE	R	ρ	OF
GEP								
	Training	4.0853	5.9697	0.0339	0.1232	0.9829	0.0622	0.0695
	Validation	5.3973	7.1330	0.0535	0.1473	0.9813	0.0743	
MEP								
	Training	2.3142	9.2813	0.0820	0.1916	0.9631	0.0976	0.1041
	Validation	2.4667	10.4115	0.1139	0.2149	0.9607	0.1085

**Table 6:** External validation.

Model	k	k'	R^2^	R_o_^2^	R_m_	m
GEP	0.972759	1.015978	0.945634	0.981071	0.767619	−0.03748
MEP	0.916793	1.06461	0.859696	0.902441	0.681955	−0.04972

### 4.3 Comparison of GEP and MEP with MLR

**Fig. 18:**
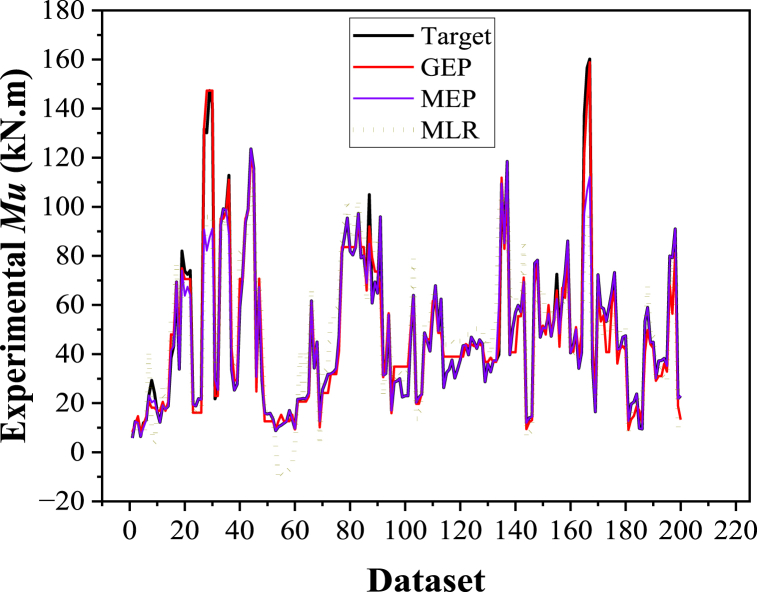
Comparison of ML models with MLR model.

To assess the accuracy of the proposed ML models, a comparison is made with the multi-linear regression (MLR) model. MLR is a statistical approach utilized to create a linear correlation between two or more independent attributes and a dependent attribute. When multiple explanatory variables are relevant, MLR is preferred over simple linear regression. Equation (10) presents the formulation for the MLR for the *M*_*u*_ property of the FRP-strengthened RC beam. Subsequently, the output attained from the MLR model is compared with those from the GEP and MEP models, as shown in Fig. 18.

(10) $$\begin{array}{c} M_{u}=‐126.908+0.2469\left(f_{c}\right)+0.0325\left(f_{y}\right)+0.00746\left(f_{f}\right)\;‐\;0.0758\left(E_{f}\right)+24.7027\left(\rho_{st}\right)\;\\ +9.3164\left(\rho_{sc}\right)+15.7064\left(\rho_{f}\right)+0.1544\left(b\right)+0.4086\left(h_{o}\right)\;‐\;0.431\left(T\right) \end{array}$$

The comparison between the experimental and estimated values of the GEP, MEP, and MLR models for the ultimate flexural capacity (*M*_*u*_) is depicted in Fig. 18. Both ML models performed exceptionally well in forecasting the *M*_*u*_. However, the GEP model demonstrated higher precision in predicting flexure strength than the MEP and MLR models. The developed GEP model exhibited exceptional performance during the validation stage and outperformed the MEP and MLR-generated models, indicating that the GEP model possesses significant prediction potential, particularly when working with unseen data. For instance, the RMSE_training_ of the GEP model is approximately 31 % and 52 % lower than that of the MEP and MLR models, respectively. On the other hand, the MLR model fails to capture the greater values for the *M*_*u*_ property adequately. This limitation hinders the practical applicability of regression approaches for predictive modeling applications.

### 4.4 Comparison with empirical models

In this part, the advanced capabilities of the ML-based estimation models are further emphasized through a comparison with empirical models. Various empirical models were employed by Zhang et al. [133] to forecast the flexural capacity of RC beams, as shown in Table 7. While certain formulas can evaluate NSM and EB methods, others particularly apply to the EB method. The predicted results obtained from these formulas were compared against the predictions generated by GEP and MEP models. Furthermore, Table 7 provides detailed information on the performance indicators and their respective values.

**Table 7:** Empirical models performance indicators.

Model	Method	Statistical indicators			Improvements in performance of the developed GEP model compared to the ACI 440.2R-08
		R	RMSE (kN.m)	MAE (kN.m)	
GB50367-2013	EB	0.9186	14.0762	9.4839	
JSCE 2001	EB	0.8808	16.8692	12.0744	
CNR-DT 200 R1	EB	0.9189	14.0585	9.9542	
TR 55	EB	0.8859	16.5275	11.8304	
ACI 440.2R-08	EB	0.9347	12.6614	8.8431	
	NSM	0.8860	12.1816	12.1816	
CSA S806-12	EB	0.9133	14.5115	10.2079	
	NSM	0.8113	15.3562	12.5057	
GEP (This study)	Training	0.9829	5.9697	4.0853	R increased by 5 % MAE decreased by 54 % RMSE decreased by 51 %
	Validation	0.9813	7.1330	5.3973	R increased by 5 % MAE decreased by 39 % RMSE decreased by 41 %

Analyzing both tables 5 and 7 present that empirical models demonstrate an R-value ranging from approximately 0.811 to 0.935, whereas ML-based models reveal a higher range of 0.960–0.983. The RMSE values for empirical models range from approximately 12 to 17 kN m, while GEP models show a lower RMSE of approximately 6–7 kN m. Similarly, the MAE values for empirical models range from approximately 8 to 13 kN m, whereas the GEP model demonstrates a lower MAE of about 4–5 kN m. Notably, the ACI 440-based EB technique demonstrates the highest prediction efficiency with an R-value of 0.935, while the CSA S806-12-based NSM technique exhibited the lowest accuracy with an R-value of only 0.811.

Furthermore, the GEP-based model's performance indicators are given in tables 5 and 7. In the training phase, the GEP model achieved an R-value of 0.983, an RMSE of 5.9697 kN m, and an MAE of 4.0853 kN m. Both the GEP model validation and training phase's performance are comparably best, indicating that the GEP-based model outperformed the empirical models significantly. Compared to the excellent empirical model (ACI 440.2R-08), the effectiveness of GEP is much superior, with R increasing by 5 %, RMSE decreasing by 51 %, and MAE decreasing by 54 % in the training set. In the validation set, R increased by 5 %, RMSE decreased by 41 %, and MAE decreased by 39 %. Overall, the results indicate that ML-based prediction models show higher accuracy levels than those based on empirical formulas.

### 4.5 Comparison with related work

The performance of the developed models was compared with the existing models in the literature for the flexural strength of FRP-strengthened beams. Murad et al. [134] utilized the GEP approach to estimate the flexural strength of the FRP-reinforced beam. Although their GEP model achieved a higher value R (0.979) compared to ACI, the model produced higher errors in terms of MAE (15.23) and RMSE (19.57) compared to ACI formulation. For an accurate prediction model, along with a higher R, the errors should also be minimal. Thus, relying solely on R as an assessment indicator for the generated model is not recommended. When comparing their GEP and ACI models, the ACI model was deemed more precise due to its similar correlation coefficients and lower error values. Furthermore, the disparity in accuracy between the GEP model and the one proposed by Murad et al. [134] can be attributed to variations in the model's parameter configurations. In addition to this, it is important to note that Murad et al. [134] employed a prediction model for beam flexural strength with six input parameters, while our approach integrated ten parameters. These differences in both model settings and the number of input variables underline the unique and tailored nature of the present study GEP model, allowing it to address the complexities and nuances of the problem with a broader information base.

In contrast, the GEP model in the present study outperformed the ACI prediction accuracy, and the MEP model produced comparable, accurate outcomes to ACI. Furthermore, Amin et al. [26] developed models based on gradient boosting trees (GBT) and decision trees (DT). The GBT model estimates the flexural strength of the FRP-strengthened beam with R, MAE, and RMSE values of 0.964, 11.74, and 15.67, respectively. These analyses provided that their model also produces a less accurate accuracy model compared to ACI. Zhang et al. [133] also developed four ensemble models, among which the gradient boosting decision tree (GBDT) model provided excellent performance. Compared to the ACI empirical model, the GBDT model exhibited significantly superior performance. The GBDT model provided notable improvements, with an increase of 9.04 % in the R^2^ value, a decrease of 31.77 % in RMSE, and a decrease of 43.97 % in MAE within the testing dataset.

## 5 Enhanced interpretability with SHAP

Previous research lacked the inclusion of machine learning interpretability techniques to elucidate the process by which predictions are made. However, as models grow more complex, there is a need for post-hoc explanations. Consequently, this study incorporated SHAP explanations to uncover the underlying rationale behind predictions and understand the contribution and influence of each variable on the output property. The SHAP concept shares similarities with parametric analysis, wherein the other variables are maintained at constant values while a specific variable is varied to observe its impact on the target attribute. Due to the superior performance of the GEP model compared to the MEP model, the GEP model was selected for SHAP analysis.

**Fig. 19:**
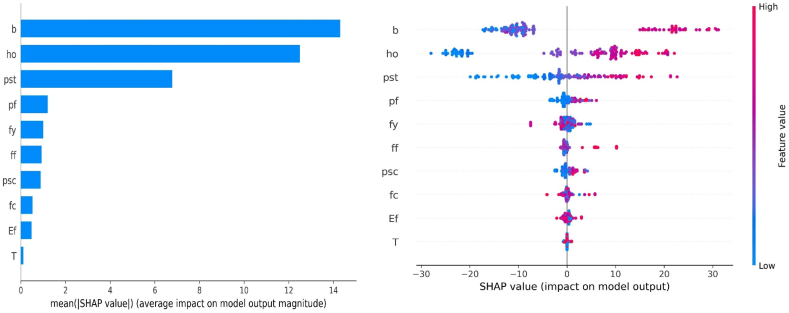
SHAP global interpretation: (a) SHAP feature importance plot, (b) SHAP summary plot.

Various approaches are available to interpret the results obtained from the prediction model. One such approach involves assessing the importance of input features to understand the influence of different attributes on the estimations of the target output. This assessment is depicted in Fig. 19a, where the results are obtained by calculating the average Shapley values across the entire dataset. It can be observed that beam width (*b*), the effective depth of section (*h*_*o*_), and the reinforcement ratio of tensile longitudinal bars (*ρ*_*st*_) significantly contribute to the prediction of flexural strength of the FRP-strengthened beam, as illustrated in Fig. 19a. The rest of the input features manifest the least contribution to the output prediction. Furthermore, it is noteworthy that the combined average absolute SHAP value of *b*, *h*_*o*_, and *ρ*_*st*_ accounts for approximately 83.13 % of the total SHAP value and represents five times greater significance than the average SHAP value of the remaining input variables.

Furthermore, Fig. 19b shows the summary plot of the SHAP analysis, which depicts the impact of each input attribute on the flexural strength (*M*_*u*_). The SHAP summary plot illustrates the correlation between the input parameters utilized in the study and their respective importance, with the y-axis representing the variables in order of significance. The x-axis of the graph represents the SHAP values, which show the influence of each variable. The colour of the dots on the graph corresponds to the magnitude of the SHAP values, ranging from small (blue) to large (red). Each dot represents a sample from the database. The horizontal x-axis illustrates the range of predictions based on the SHAP values for each parameter, showing the varying impact of input attributes from blue to red. The width of the beam positively influences the flexural strength of the beam, indicating that increasing the width of the beam will result in increased *M*_*u*_. For instance, Amin et al. [26] reported that raising the width from 130 mm to 381 mm caused a significant increase of approximately 6 kN m in the bending capacity.

Similarly, the same trend can be noted in the values of the effective depth of the beam (*h*_*o*_) such that flexural capacity increases with the *h*_*o*_. The concepts of empirical equations and mechanics indicate a comparable relationship between the change in beam depth and variations in flexural capacity. It can also be noticed that the increased reinforcement ratio of tensile longitudinal bars (*ρ*_*st*_) also favourably affects the *M*_*u*_ of the FRP-strengthened RC beam. Furthermore, the input attributes associated with the properties of FRP make a lesser contribution, which is understandable considering that the capacity supplied by FRP is minor when compared to the experimental bearing capacity of the RC beam.

## 6 Limitations of the study and recommendation for future work

The present work utilized the GEP and MEP models to predict the flexural capacity of FRP-strengthened RC beam in the range of 5.79–160.2 kN m range. However, it is highly recommended to utilize various metaheuristic optimization techniques such as Particle Swarm Optimization (PSO), Grey Wolf Optimization (GWO), and the Human Felicity Algorithm (HFA), as these methods have the potential to yield more accurate results for predicting the flexural capacity of FRP-strengthened RC beam. Furthermore, the SHAP global interpretation is used in the current study. However, the local interpretability of the ML model based on Partial Dependence Plots (PDP) and SHAP techniques can be used for future models. Furthermore, the current study utilized the database, containing only 200 data points, which is very limited. Therefore, an enhanced database is essential to bolster the empirical foundation of the models, ensuring a more comprehensive representation of real-world conditions and scenarios. A diverse and enriched dataset will improve the reliability and applicability of the predictive models for flexural capacity in FRP-strengthened RC beams, thereby enhancing their practical utility in engineering applications.

## 7 Conclusion

This study proposes model-based ML methods to forecast the flexural capacity of FRP-strengthened RC beams. A database consisting of 200 sets of experimental data was compiled from experimental studies for training and validating models. Using the collected database, prediction models for flexural capacity were developed using GEP and MEP algorithms empirical estimation models. The models' performance and accuracy were evaluated using a range of statistical indicators. To explain the models, SHAP was utilized to analyze the contribution of each feature and direction toward the prediction outcome. The findings of this work can be briefly provided as follows.

1. In general, the flexural capacity prediction models using the ML approaches demonstrated excellent accuracy. The models achieved an R score above 0.96 in both the validation and training phases. Among both models, GEP showed superior predictions, with R, RMSE, and MAE scores of 0.981, 7.133, and 5.39, respectively, on the validation set. The majority of the predicted records in the GEP model aligned well with the experimental records. The models performed well on both sets (validation, training), with RMSE and MAE values at lower levels. This indicates that the models provided strong generalization and high accuracy capabilities.
2. The accuracy of ML-based prediction models, particularly GEP, provided superior accuracy than empirical models. In contrast to the best-performing empirical models, the GEP-based model exhibited significant improvements. In the validation phase, the GEP-based model achieved a 5 % increase in R, a 41 % decrease in RMSE, and a 39 % decrease in MAE compared to the ACI 440.2R-08. These findings support that ML-based prediction models could be viable alternatives to empirical models to estimate the output accurately.
3. The SHAP analysis reveals that beam depth (*b*), the effective depth of section (*h*_*o*_), and the reinforcement ratio of tensile longitudinal bars (*ρ*_*st*_) have the most substantial positive impact on the predicted output, while the parameters associated with the properties of FRP exhibited comparatively less contribution.

## Data availability

Data will be made available on request.

## Additional information

No additional information is available for this paper.

## CRediT authorship contribution statement

**Majid Khan:** Visualization, Validation, Formal analysis. **Adil Khan:** Writing - original draft. **Asad Ullah Khan:** Writing - original draft, Visualization, Validation. **Muhammad Shakeel:** Supervision, Software, Formal analysis, Data curation. **Khalid Khan:** Writing - review & editing, Writing - original draft, Supervision, Software, Conceptualization. **Hisham Alabduljabbar:** Writing - review & editing, Supervision, Funding acquisition. **Taoufik Najeh:** Resources, Funding acquisition. **Yaser Gamil:** Writing - original draft, Software, Project administration.

## Declaration of Competing interest

The authors declare that they have no known competing financial interests or personal relationships that could have appeared to influence the work reported in this paper.
